# Effect of Meloxicam on the Pharmacokinetics of Cefquinome in Endotoxemic Sheep

**DOI:** 10.1002/vms3.70462

**Published:** 2025-09-04

**Authors:** Muhittin Uslu, Orhan Corum, Enver Yazar

**Affiliations:** ^1^ Department of Laboratory and Veterinary Health Sefaatli Vocational College University of Yozgat Bozok Yozgat Türkiye; ^2^ Department of Pharmacology and Toxicology Faculty of Veterinary Medicine University of Hatay Mustafa Kemal Hatay Türkiye; ^3^ Department of Pharmacology and Toxicology Faculty of Veterinary Medicine University of Selcuk Konya Türkiye

**Keywords:** cefquinome, endotoxemic sheep, meloxicam, MIC, pharmacokinetics

## Abstract

**Objective:**

The objective of this study was to investigate the effect of meloxicam on the pharmacokinetics of cefquinome in experimental endotoxemic sheep. In addition, the MIC of cefquinome was determined against *Escherichia coli*, *Pasteurella multocida*, *Klebsiella pneumoniae*, and *Mannheimia haemolytica*.

**Methods:**

The study was carried out on six sheep in three periods according to a longitudinal pharmacokinetic design. Cefquinome (2.5 mg/kg, IV, CFQ) was administered in the first period, cefquinome+meloxicam (1 mg/kg, IV, CFQ+MLX) in the second period, and lipopolysaccharide (20 µg/kg, IV, LPS+CFQ+MLX)+meloxicam+cefquinome in the third period. Plasma cefquinome concentrations were assayed using HPLC‐UV, and pharmacokinetic data were calculated by a two‐compartment open model.

**Results:**

Following a single IV injection of cefquinome, the t_1/2β_, V_dss_, and Cl_T_ values were 1.12 h, 0.21 L/kg, and 0.17 L/h/kg, respectively. The t_1/2β_ was prolonged from 1.12 to 2.79 h in the LPS+CFQ+MLX group. While V_dss_ was increased (from 0.21 to 0.36 L/kg) in the LPS+CFQ+MLX group, Cl_T_ decreased (from 0.17 to 0.10 L/h/kg) in the CFQ+MLX and LPS+CFQ+MLX groups. The MICs of cefquinome were 0.031 to 0.063 µg/mL for *E. coli, M. haemolytica*, and *K. pneumoniae* and 0.016 to 1 µg/mL for *P. multocida*. At a 12 h dosing interval, the CFQ, CFQ+MLX, and LPS+CFQ+MLX groups attained a T > MIC ratio of 40% for bacteria with MIC values of ≤ 0.50, ≤ 1, and ≤ 1 µg/mL, respectively.

**Conclusion:**

These results indicate that combined administration of meloxicam alters the pharmacokinetics and therapeutic efficacy of cefquinome in experimental endotoxemic sheep.

## Graphical abstract

Pharmacokinetics of cefquinome (2.5 mg/kg, CFQ) after intravenous administration alone and co‐administered with meloxicam (MLX) or MLX + LPS in sheep.

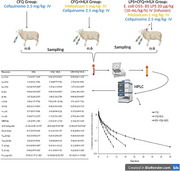

## 1 Introduction

The presence of lipopolysaccharides (LPS), the most important bacterial antigen in the cell wall structure of gram‐negative bacteria, in the bloodstream is called endotoxemia (Bochud and Calandra 2003; López‐Bojórquez et al. 2004). Endotoxemia has been associated with Gram‐negative bacteria, including *Escherichia coli*, *Pasteurella multocida*, *Mannheimia haemolytica*, and *Klebsiella spp*. (Bochud and Calandra 2003; Jesse et al. 2019). Endotoxemia causes systemic inflammation due to the synthesis and release of inflammatory mediators such as histamine, kinin, prostaglandin, platelet‐activating factor, serotonin, and proinflammatory cytokines (Kelmer 2009; Park and Lee 2013). Depending on these changes, pathophysiological changes such as decreased white blood cell count, increased body temperature and haemodynamic alterations are observed (Blatteis and Sehic 1998; van Lier et al. 2019). The use of antibiotics and non‐steroidal anti‐inflammatory drugs (NSAIDs) is recommended in the treatment of endotoxemia to prevent the release of endotoxins and reduce inflammatory mediators (Moore and Barton 2003).

Cefquinome is a fourth‐generation cephalosporin antibiotic with a broad spectrum of activity that has been approved for use in veterinary medicine. It is classified as category B (Restrict) by the European Medicines Agency (EMA) in terms of the risk of antimicrobial resistance (EMA 2020, Papich 2016). It shows bactericidal activity by inhibiting the cell wall synthesis of sensitive bacteria. While cefquinome is highly effective against Gram‐negative and some anaerobic (*Clostridium spp*., etc.) bacteria, its effectiveness against Gram‐positive bacteria is low (Papich 2016; Yazar 2018, 2021). It is also highly effective against β‐lactamases encoded by genes on chromosomes and plasmids (Durna Corum et al. 2022; Limbert et al. 1991). Cefquinome is approved for use in cases of respiratory, urinary and digestive system infections, foot infections, metritis, mastitis and septicemia in animals such as cattle and horses (CVMP 1999, 2003; Yazar 2018). Although cefquinome is not licensed for sheep, it can be used off‐label in these disease states. The pharmacokinetics of cefquinome in different animals (Elbadawy et al. 2021; El‐Hewaity et al. 2014; Uney et al. 2018) and sheep (Corum et al. 2019a; Tohamy 2011; Uney et al. 2011) have been studied and these investigations are crucial for posological optimisation (EMA 2017).

Meloxicam is a long‐acting NSAID of the oxicam group with anti‐inflammatory, analgesic, antiexudative and antipyretic effects (Davies and Skjodt 1999; Yazar 2018). Meloxicam is approved for use in cattle, horses, pigs, dogs, cats and guinea pigs, and it has been stated that its use in sheep has been approved in Canada, Australia and New Zealand, and many studies have been conducted in sheep (CVMP 2006; Coskun et al. 2023; Gungor et al. 2024; de la Puente et al. 2024). Meloxicam's mechanism of action relies on inhibiting the activity of the cyclooxygenase (COX) enzyme, which is responsible for synthesising prostaglandins from arachidonic acid. Since meloxicam has an inhibitory effect, especially on the COX‐2 enzyme, its side effects on the gastrointestinal system are low (Coskun et al. 2023; Gates et al. 2005; Woodland et al. 2019). NSAIDs are effective in endotoxemia because they reduce prostaglandin and thromboxane production (Langston et al. 1995), and meloxicam provided clinical improvement in endotoxemic pigs (Friton et al. 2006; Wyns et al. 2015).

Simultaneous use of two drugs may cause pharmacokinetic drug interactions, in which case the effectiveness of the drugs may change (Altan et al. 2020; Durna Corum et al. 2020). The effect of cefquinome is time‐dependent, and the pharmacokinetic/pharmacodynamic parameter used to evaluate its antibacterial effect is the percentage of time interval (T > MIC%) that plasma concentration remains above the MIC (Corum et al. 2019a). This parameter is obtained using the apparent volume of distribution (V_darea_), terminal elimination half‐life (t_1/2ʎz_) and MIC (Corum et al. 2019a). Therefore, changes in the pharmacokinetics of cefquinome also affect the success of the treatment. Simultaneous administration of meloxicam with marbofloxacin and ceftriaxone caused changes in pharmacokinetic parameters such as V_darea_, t_1/2ʎz_ and total body clearance (Gond et al. 2023; Ranjan et al. 2012; Ural and Uney 2021). Meloxicam can be used simultaneously with cefquinome in the treatment of endotoxemia. To our knowledge, while the impact of meloxicam on the pharmacokinetics of cefquinome has been determined in camels (Kant et al. 2024), no research has been found in sheep. The aim of this study is (a) to investigate the effect of meloxicam on the intravenous (IV) pharmacokinetics of cefquinome (2.5 mg/kg) in experimental endotoxemic sheep; (b) to determine the minimum inhibitory concentration (MIC) of cefquinome against *E. coli*, *P. multocida*, *M. haemolytica*, and *K. pneumoniae* isolated from sheep; and (c) to establish the pharmacokinetic‐pharmacodynamic relationship of cefquinome in the treatment of endotoxemia using the pharmacokinetic and MIC values obtained from this study.

## 2 Materials and Methods

### 2.1 Animals

Six healthy Merino sheep (1.71 ± 0.27 years old, 57.33 ± 5.12 kg) were utilised. The sheep were evaluated as healthy by a complete blood count and a general physical examination. None of the animals selected for the study were administered any medication during the last 30 days. The sheep were placed in pens seven days before the study for the acclimatisation period. The sheep were fed commercial feed, and water and hay were available ad libitum. The study protocol was approved (2023/012) by the Ethics Committee of the Faculty of Veterinary Medicine, University of Selcuk.

### 2.2 Experimental Design

Before the study, a catheter (18 G, 1.3 mm × 45 mm) was placed in the sheep for blood collection (right jugular vein) and drug administration (left jugular vein). The study was carried out in three periods according to the longitudinal pharmacokinetic design. When each stage was completed, a 15‐day drug washout period was applied before moving on to the next period. In the first period (CFQ), cefquinome (2.5 mg/kg, Cobactan, 2.5%, Intervet) was administered to the sheep intravenously. In the second period (CFQ+MLX), first meloxicam (1 mg/kg, IV, Maxicam, Sanovel) was administered via a catheter into the left jugular vein, followed by cefquinome (2.5 mg/kg, IV, Cobactan, 2.5%, Intervet) within one minute. In the third period (LPS+CFQ+MLX), following the administration of LPS as a 1 h infusion, first meloxicam was administered, and then cefquinome was administered within one minute, as in the second period. The endotoxemia model was established by administering E. coli O55: B5 LPS (Sigma‐Aldrich, USA) dissolved in saline to sheep as a 1 h IV infusion (10 mL/kg/h) at a dose of 20 µg/kg (Chalmeh et al. 2013). Blood samples were obtained using a catheter within the first 12 h and by the venipuncture method at other sampling times. For pharmacokinetic analysis, blood samples (1 mL) were obtained (right jugular vein) into tubes containing lithium heparin at 0 (control), 0.08, 0.17, 0.25, 0.33, 0.5, 0.75, 1, 1.5, 2, 3, 4, 5, 6, 7, 8, 10, 12, 18, 24, 48, and 72 h. Blood samples were centrifuged (4,000 g, 10 min) to obtain plasma samples and stored at −80°C until cefquinome analysis. Haematological analysis and rectal temperature measurements were performed to confirm the accuracy of the endotoxemia model in the LPS group. For hemogram analysis, blood samples (1 mL) were obtained into tubes containing K3‐EDTA at ‐1 (before LPS administration), 0 (before drug administration), 0.25, 0.5, 1, 2, 3, 4, 8, 12, 24, 48, and 72 h. Additionally, rectal temperature was measured at these times. Haematological parameters were examined immediately after collecting blood samples.

### 2.3 Cefquinome Analysis

Cefquinome analysis from plasma samples was measured using an HPLC‐UV method previously reported (Uney et al. 2011). Briefly, plasma samples stored at ‐80°C were allowed to reach room temperature. The 400 µL of methanol was added to a microcentrifuge tube containing 200 µL of plasma. The vortexed (30‐sec) samples were centrifuged at 12,500 g for 10 min to obtain the supernatant. Then, 300 µL of supernatant was placed into a new microcentrifuge tube, and 150 µL of ultrapure water was added. The mixed samples were put into vials, and 50 µL was injected into the HPLC. The HPLC system (Shimadzu/Japan) was equipped with a SPD‐20A UV‐VIS detector, a CTO‐10A column oven, a DGU‐20A degasser, a SIL‐20A auto‐sampler, and a LC‐20AT pump. The HPLC separation of cefquinome was carried out with an inert Sustain C18 analytical column (250×4.6 mm; 5 µm particle size), maintained at 40°C. The wavelength was set to 268 nm. The mobile phase with a flow rate of 1 mL/min consisted of 0.1% trifluoroacetic acid (A) and acetonitrile (B). The gradient elution technique was 0–7 min, 90% A; 7–15 min, 50% A; and 15–16 min, 90%.

The chromatographic method was validated according to the European Medicines Agency guidelines (EMA 2011). The cefquinome stock solution was prepared in ultrapure water to achieve a concentration of 1 mg/mL. Calibration standards (0.02‐40 µg/mL) and quality control (QC) samples (0.05, 1, and 10 µg/mL) were prepared in blank sheep plasma. To ascertain precision, accuracy, and recovery, QC samples of cefquinome were evaluated in six replicates over a span of six days. Cefquinome recoveries were determined by comparing QC samples with working standards. The precision was assessed using the coefficient of variation (CV), while accuracy is quantified as bias [Bias (%) = 100 times (calculated concentration−theoretical concentration)/theoretical concentration].

### 2.4 Pharmacokinetic Analysis

Pharmacokinetic parameters were calculated individually using WinNonlin 6.1. software. The fit of the pharmacokinetic model was determined by visual inspection of individual concentration‐time curves and application of Akaike's information criterion (Yamaoka et al. 1978). The pharmacokinetic parameters of cefquinome in each animal were fitted to a two‐compartment open model.

### 2.5 Determination of Minimal Inhibitory Concentrations

The broth microdilution method was employed to ascertain the MICs of cefquinome for bacteria (*E. coli, P. multocida, K. pneumoniae*, and *M. haemolytica*). Bacterial clinical strains isolated from sheep in the culture collection of Selcuk University Faculty of Veterinary Medicine Microbiology laboratory in 2023—2024, when the study was conducted and confirmed as diagnostic pathogens biochemically and molecularly, were used. The microdilution antimicrobial susceptibility tests were carried out as stated in the Clinical and Laboratory Standards Institute (CLSI) guidelines (CLSI 2024). All strains with McFarland 0.5 standard turbidity were passaged 10^5^ CFU bacteria. A series of 12 different concentrations of cefquinome ranging from 0.0039 to 2 µg/mL were prepared in Mueller Hinton broth. Subsequently, 100 µL of each concentration was added to individual wells of a 96‐well plate for the purpose of conducting the MIC test. Turbidities were evaluated with an ELISA reader compared with control wells, and the lowest cefquinome concentration at which bacterial growth was inhibited was considered the MIC value, as stated in the CLSI 2024 data (CLSI, 2024). To verify the minimum inhibitor concentration, the ELISA reader was cultured from the well where there was no bacterial growth with the same OD as the control group and checked for bacterial growth.

### 2.6 Pharmacokinetic/Pharmacodynamic Integration

The T>MIC value of cefquinome was performed using the pharmacokinetic parameters obtained in this study and the MIC value determined for *E. coli, P. multocida, K. pneumoniae*, and *M. haemolytica* bacteria isolated from sheep. The T>MIC value was calculated according to the previously mentioned formula (Corum et al. 2019b).

### 2.7 Hemogram Analysis

Haemogram parameters such as white blood cell (WBC), red blood cell (RBC), platelet, haemoglobin, and haematocrit were analysed using a blood cell counter (Auto Haematology Analyzer, BC‐2800, Mindray) immediately after blood collection.

### 2.8 Statistical Analysis

Haemogram parameters and rectal temperature values are presented as mean±SD, and statistical differences were evaluated using one‐way analysis of variance and post hoc Tukey tests (SPSS 22.0, IBM Corp.). Pharmacokinetic parameters are reported as geometric mean (minimum‐maximum), and statistical differences between treatment groups were assessed using the Mann‐Whitney U test. p < 0.05 was considered to be statistically significant.

## 3 Results

### 3.1 Physiological Parameters

**FIGURE 1:**
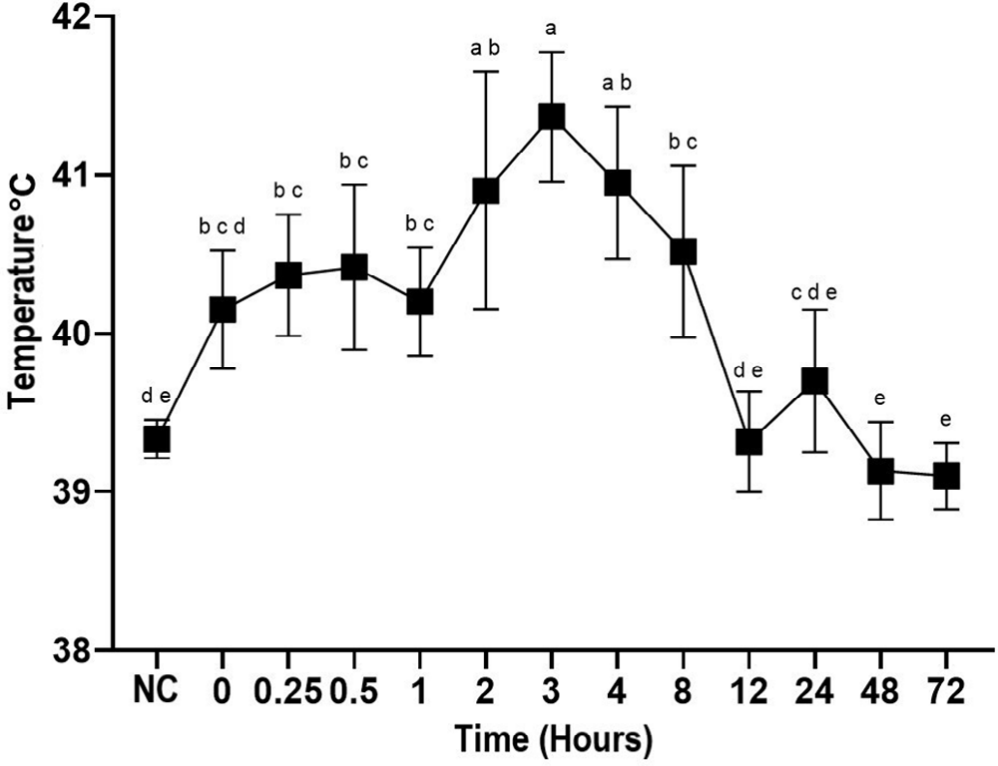
Rectal temperature (°C) after intravenous administration (2.5 mg/kg) of cefquinome co‐administered with meloxicam + LPS in sheep (n = 6, mean ± SD). Different letters (^a, b, c, d, e^) between times indicate statistical significance (P < 0.05).

**FIGURE 2:**
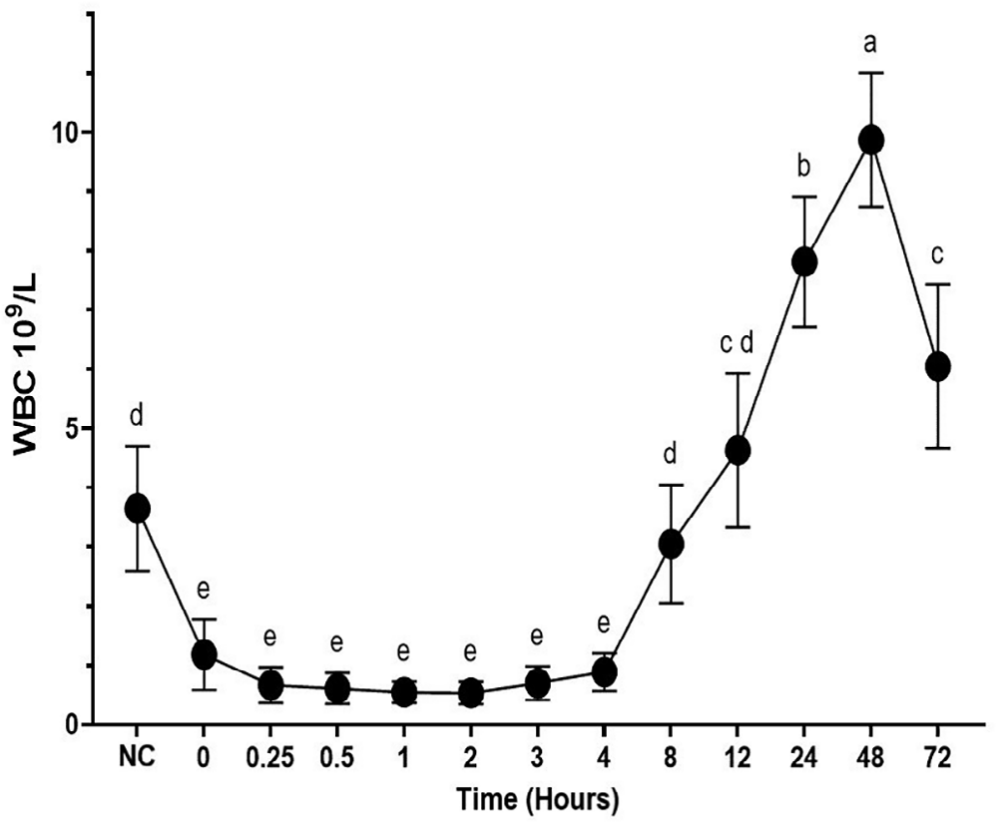
White blood cell (WBC) counts after intravenous administration (2.5 mg/kg) of cefquinome (CFQ) co‐administered with meloxicam + LPS in sheep (n = 6, mean ± SD). Different letters (^a, b, c, d, e^) between times indicate statistical significance (P < 0.05).

In the LPS+ CFQ+MLX group, rectal temperature increased between 0.25‐8 h (P < 0.05, Figure 1), while WBC value decreased between 0–4 h (P < 0.05, Figure 2). Statistical fluctuations were also observed in RBC, platelet, haemoglobin, and haematocrit values (P < 0.05, Table 1).

**TABLE 1:** Hematological parameters after intravenous administration (2.5 mg/kg) of cefquinome co‐administered with meloxicam + LPS in sheep (n = 6, mean ± SD).

	Time (hours)												
Parameters	NC	0	0.25	0.5	1	2	3	4	8	12	24	48	72
WBC (10^9^/L)	3.64±1.05^d^	1.18± 0.59^e^	0.66±0.30^e^	0.60±0.26^e^	0.54±0.18^e^	0.53±0.18^e^	0.69±0.28^e^	0.88±0.31^e^	3.04±1.00^d^	4.62±1.29^cd^	7.81±1.09^b^	9.87±1.13^a^	6.04±1.38^c^
RBC (10^12^/L)	12.53±1.66^ab^	14.01±1.29^a^	12.59±1.43^ab^	11.63±1.95^ab^	11.75±1.43^ab^	12.99±1.79^ab^	13.52±1.97^ab^	13.41±2.00^ab^	12.26±0.99^ab^	11.39±1.23^ab^	11.26±0.86^ab^	10.91±1.06^b^	10.86±1.84^b^
PLT (10^9^/L)	250.50±27.37^cdef^	287.16±46.39^bcde^	320.50±22.09^abc^	330.00±39.10^abc^	341.00±29.50^ab^	401.00±103.61^a^	302.16±39.46^bcd^	300.00±55.46^bcd^	220.00±38.43^def^	172.16±24.70^f^	185.00±22.15^f^	183.16±33.34^f^	208.66±30.03^ef^
HG (g/dL)	11.55±1.08^abcd^	13.11±0.73^a^	11.63±0.78^abc^	10.71±1.39^bcde^	10.85±0.87^bcde^	11.51±1.00^abcd^	12.33±1.14^ab^	12.23±1.09^ab^	11.60±0.51^abc^	10.51±0.65^bcde^	10.30±0.37^cde^	9.71±0.49^de^	9.55±1.23^e^
HCT (%)	39.46±5.07^ab^	44.68±1.94^a^	40.06±3.65^ab^	36.53±5.36^b^	36.81±2.55^b^	41.01±3.83^ab^	42.65±4.17^ab^	42.63±4.86^ab^	39.65±2.41^ab^	35.68±2.74^b^	35.66±2.31^b^	34.95±2.70^b^	35.43±6.20^b^

Abbreviations: WBC, white blood cell; RBC, red blood cell; PLT, platelet; HB, haemoglobin; HCT, haematocrit; NC, negative control.

^a,b,c,d,e,f^: Varied characters in the same row are statistically different (p < 0.05).

### 3.2 Method Validation

The lack of plasma and other origin peaks during the retention duration of cefquinome on the chromatogram indicates that this approach possesses excellent specificity. The calibration curve for cefquinome exhibited a high degree of linearity (R^2^> 0.9990) across the concentration range of 0.02–40 µg/mL. Cefquinome recovery from plasma was ≥ 90%. The lower limit of quantification of cefquinome in sheep plasma was 0.02 µg/mL, with a bias of ±15% and a CV below 20%. The limit of detection for plasma was 0.01 µg/mL. The CV for intra‐day and inter‐day measurements were ≤ 4.80% and ≤ 6.20%, respectively. The intraday bias had a range of ± 6.6%, while the interday bias had a range of ± 8.6%.

### 3.3 Pharmacokinetic Parameters

**FIGURE 3:**
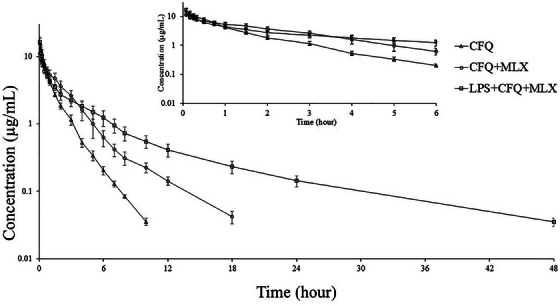
Semi‐logarithmic plasma concentration‐time curve of cefquinome (2.5 mg/kg, CFQ) after intravenous administration alone and co‐administered with meloxicam (MLX) or MLX + LPS in sheep (n = 6, mean ± SD).

Semi‐logarithmic plasma concentration‐time curves and pharmacokinetic parameters of cefquinone are presented in Figure 3 and Table 2. Cefquinome was detected up to 10, 18 and 48 h in the CFQ, CFQ+MLX and LPS+CFQ+MLX groups, respectively. After a single administration of cefquinome, t_1/2α_, t_1/2β_, V_dss_ and Cl_T_ values were 0.17 h, 1.12 h, 0.21 L/kg and 0.17 L/h/kg, respectively. While t_1/2β_ and V_dss_ were increased in the LPS+CFQ+MLX group, Cl_T_ decreased in the CFQ+MLX and LPS+CFQ+MLX groups (P > 0.05). The t_1/2α_ and V_darea_ were similar in all groups (P < 0.05). Compared to the CFQ and CFQ+MLX groups, the k_21_/k_12_ ratio decreased and the k_12_/k_21_ ratio increased in the LPS+CFQ+MLX group.

**TABLE 2:** Plasma pharmacokinetic parameters of cefquinome (2.5 mg/kg, CFQ) after intravenous administration alone and co‐administered with meloxicam (MLX) or MLX + LPS in sheep (n = 6).

Parameters	CFQ	CFQ +MLX	LPS+CFQ+MLX
k_10_ (1/h)	1.33 (0.97‐1.80)^a^	0.68 (0.58‐0.93)^b^	0.89 (0.61‐1.18)^b^
k_12_ (1/h)	1.10 (0.21‐3.58)	2.49 (0.77‐5.52)	2.58 (1.94‐4.93)
k_21_ (1/h)	1.83 (0.39‐5.92)^ab^	4.15 (2.14‐6.59)^a^	1.27 (1.04‐1.65)^b^
k_12_/k_21_	0.60 (0.44‐0.81)^b^	0.60 (0.36‐0.90)^b^	2.04 (1.46‐2.99)^a^
k_21_/k_12_	1.67 (1.24‐2.30)^a^	1.66 (1.11‐2.76)^a^	0.49 (0.33‐0.69)^b^
α (1/h)	3.96 (1.37‐9.78)	7.02 (3.09‐ 12.44)	4.54 (3.63‐7.33)
β (1/h)	0.62 (0.31‐0.98)^a^	0.40 (0.36‐0.46)^a^	0.25 (0.22‐0.31)^b^
*t*_1/2α_ (h)	0.17 (0.07‐0.50)	0.10 (0.06‐0.22)	0.15 (0.09‐0.19)
*t*_1/2β_ (h)	1.12 (0.71‐2.27)^b^	1.73 (1.50‐1.93)^b^	2.79 (2.26‐3.19)^a^
MRT (h)	1.21 (0.95‐1.54)^c^	2.39 (2.05‐ 2.72)^b^	3.44 (2.54‐4.13)^a^
AUC (h*µg/mL)	14.46 (12.90‐16.17)^b^	20.94 (16.35‐25.79)^a^	23.88 (18.70‐27.61)^a^
Cl_T_ (L/h/kg)	0.17 (0.15‐0.19)^a^	0.12 (0.10‐0.15)^b^	0.10 (0.09‐0.13)^b^
V_dss_ (L/kg)	0.21 (0.16‐0.27)^c^	0.28 (0.25‐0.31)^b^	0.36 (0.32‐0.41)^a^
V_darea_ (L/kg)	0.28 (0.19‐0.54)	0.30 (0.26‐0.33)	0.42 (0.38‐0.47)
V_1_ (L/kg)	0.13 (0.09‐0.19)^ab^	0.18 (0.14‐0.21)^a^	0.12 (0.09‐0.15)^b^
V_2_ (L/kg)	0.08 (0.07‐0.10)^b^	0.11 (0.08‐0.15)^b^	0.24 (0.21‐0.29)^a^
C_0.08_ (µg/mL)	15.38 (11.37‐21.48)^a^	11.08 (10.42‐12.38)^b^	16.14 (13.44‐19.36)^a^

Abbreviations: k_10_, the rate of elimination from the central compartment; k_12_, rate of transfer from central to peripheral compartment; k_21_, rate of transfer from peripheral to central compartment; α, distribution rate constant; β, elimination rate constant; t_1/2α_, distribution half‐life; t_1/2β_, elimination half‐life; MRT, mean residence time; AUC, area under the plasma concentration–time curve; Cl_T_, total clearance; V_dss_, volume of distribution at steady state; V_darea_, apparent volume of distribution; V_1_, volumes of central compartments; V_2_, volumes of peripheral compartments, C_0.08 h_, plasma concentration at first sampling time.

^a,b,c^: Varied characters in the same row are statistically different (p < 0.05).

### 3.4 Pharmacokinetic–Pharmacodynamic Integration

The MIC values of cefquinome obtained for *E. coli, P. multocida, K. pneumoniae*, and *M. haemolytica* bacteria isolated from sheep are presented in Table 3. While the MIC value was between 0.031 and 0.063 µg/mL for *E. coli, M. haemolytica* and *K. pneumonia*, it was between 0.016 and 1 µg/mL for *P. multocida*. All isolated bacteria were found to be cefquinome sensitive (≤ 2 µg/mL for susceptible breakpoint, García et al. 2022). The T > MIC ratios of cefquinome calculated for strains below the susceptible breakpoint are presented in Table 4. In the CFQ, CFQ+MLX, and LPS+CFQ+MLX groups, at a 12‐h dosing interval, a T > MIC ratio of 40% was achieved for bacteria with MIC values of ≤ 0.50, ≤ 1 and ≤ 1 µg/mL, respectively. At the 8‐h dosing interval, a T > MIC ratio of 40% was achieved for bacteria with MIC values of ≤ 1, ≤ 2, and ≤ 2 µg/mL, respectively.

**TABLE 3:** MIC values (µg/mL) of cefquinome for different bacterial species isolated from sheep.

Isolates number	*E. coli*	*P. multocida*	*M. haemolytica*	*K. pneumonia*
1	0.063	1	0.063	0.063
2	0.031	1	0.031	0.031
3	−	0.031	−	−
4	−	0.016	−	−
5	−	0.125	−	−

Abbreviations: MIC, minimum inhibitory concentration.

**TABLE 4:** Calculated T > MIC% values after intravenous administration of cefquinome (CFQ) alone and co‐administered with meloxicam (MLX) or MLX + LPS in sheep.

	8 h			12 h		
MIC (µg/mL)	CFQ	CFQ+MLX	LPS+ CFQ+MLX	CFQ	CFQ +MLX	LPS+ CFQ +MLX
0.06	100	100	100	72	100	100
0.12	92	100	100	62	89	100
0.25	76	100	100	51	73	100
0.50	60	89	100	40	59	84
1	45	67	90	30	45	60
2	29	45	55	20	30	37

*Note*: Even if the T>MIC value is greater than 100%, it is expressed as 100%.

Abbreviations: CFQ, cefquinome; MLX, meloxicam, LPS, Lipopolysaccharide.

## 4 Discussion

LPS administration causes a systemic inflammatory response characterised by increased body temperature, decreased WBC, and haemodynamic changes (Moore 1988). In the LPS+CFQ+MLX group, rectal temperature of the sheep increased up to 8 h following LPS application, while WBC value decreased up to 4 h. Similar results were reported in different experimental endotoxemia models induced by LPS in sheep (Coskun et al. 2020). In addition, statistical fluctuations were detected in the RBC, platelet, haemoglobin, and haematocrit values in the LPS+CFQ+MLX group within the reference value range reported in sheep (Etim 2015). In this study, changes in rectal temperature and WBC values indicate that endotoxemia has developed in sheep.

It is recommended to use cefquinome in cattle, calves, and pigs at a dose of 1–2 mg/kg for 3–5 days (CVMP 2003). Cefquinome has been used in small ruminants at doses ranging from 1 to 20 mg/kg (Tiwari et al. 2015; Tohamy 2011; Uney et al. 2011). Cefquinome has been successfully used in sheep at a dose of 2.5 mg/kg (Corum et al. 2019a) and has not shown any adverse effects on haematological and biochemical parameters at this dose (Corum et al. 2022). Therefore, a dose of 2.5 mg/kg of cefquinome was preferred in this study.

In the study that looked at how well different amounts of meloxicam worked to treat lameness caused by turpentine in sheep, it was found that the 0.5 mg/kg dose was not very effective, while the 1–2 mg/kg doses worked similarly well, and the 1 mg/kg dose was enough for treatment (Colditz et al. 2019; Woodland et al. 2019). Therefore, this study preferred the 1 mg/kg dose of meloxicam.

The V_dss_ of cefquinome (0.21 L/kg) increased significantly in the CFQ+MLX (0.28 L/kg) and LPS+CFQ+MLX (0.36 L/kg) groups. The V_darea_ values were different between groups but were not statistically significant due to the range of values. The V_darea_ is greater than the V_dss_ for all medications (Toutain and Bousquet‐mélou 2004). The V_dss_ value of cefquinome was 0.21 L/kg, which is consistent with the value (0.28‐0.36 L/kg) previously reported in sheep (Uney et al. 2011; Corum et al. 2019a). Cefquinome has a hydrophobic structure and is found in ionised form at blood pH due to its low pKa (2.51 or 2.91) (CVMP 1995). This limits the volume of distribution of cefquinome. The V_dss_ value showed a significant difference in the order of LPS+CFQ+MLX > CFQ+MLX > CFQ. Meloxicam is highly (96‐99%) bound to plasma proteins, whereas cefquinome is lowly (8‐16%) bound (CVMP 1995, 2006; El‐Hewaity et al. 2014; Tohamy 2011). The high plasma protein binding of meloxicam may cause a further decrease in the plasma protein binding ratio of cefquinome. Therefore, due to the simultaneous use of cefquinome with meloxicam in the CFQ+MLX group, meloxicam may cause an increase in the Vd_ss_ value of cefquinome. Plasma proteins and their binding capacity decrease due to vascular haemodynamic changes that occur in the event of endotoxemia (Dickson and Lehmann 2019). The high V_dss_ in the CFQ+MLX and LPS+CFQ+MLX groups may be due to the decreased binding of cefquinome to plasma proteins.

The Cl_T_ of cefquinome (0.17 L/h/kg) decreased significantly in the CFQ+MLX (0.12 L/h/kg) and LPS+CFQ+MLX (0.10 L/h/kg) groups. Cefquinome undergoes minimal metabolism and is mostly eliminated unaltered through renal excretion (CVMP 1995; Limbert et al. 1991). Cephalosporins are excreted by glomerular filtration and tubular secretion via organic anion transporter (OAT)‐1 (Fanos and Cataldi 2001). The liver and kidney are the two major organs responsible for medication excretion, and decreasing blood supply to these organs might affect drug clearance (Morgan 2009). Drugs with a low hepatic extraction ratio are minimally affected by changes in liver blood flow rate (Yang and Lee 2008); therefore, changes in cefquinome Cl_T_ may be related to renal function. NSAIDs inhibit prostaglandin (E_2_ and I_2_) synthesis, reducing renal blood flow and glomerular filtration rate (Harirforoosh and Jamali 2009). Additionally, meloxicam inhibits OAT‐1 and 3 transporters (Zou et al. 2021). Similarly, meloxicam reduced the Cl_T_ of ceftriaxone (Ranjan et al. 2012) and danofloxacin (Ural and Uney 2021) in sheep and marbofloxacin in calves (Gond et al. 2023). Endotoxemia impairs renal function, leading to damage in the proximal tubule and a decrease in blood flow to the kidneys and glomerular filtration rate (van Lier et al. 2019). The Cl_T_ of drugs such as cefquinome (Tiwari et al. 2015), marbofloxacin (Coskun et al. 2020), and enrofloxacin (Post et al. 2003) decreased after LPS administration to animals. The Cl_T_ may have decreased in the CFQ+MLX and LPS+CFQ+MLX groups due to the reasons mentioned above.

In this study, t_1/2β_ of cefquinome was prolonged from 1.12 to 2.79 h in the LPS+CFQ+MLX group. Although t_1/2β_ was prolonged from 1.12 to 1.73 h in the CFQ+MLX group, this was not statistically significant. It has been reported that t_1/2ʎz_ is prolonged due to endotoxemia in sheep with marbofloxacin (from 2.87 to 4.64 h, Coskun et al. 2020), in pigs with enrofloxacin (from 10.5 to 16.2 h, Post et al. 2003), in camels with danofloxacin (from 5.1 to 10.2 h, Al‐Taher 2013) and in calves with ceftiofur (from 19.9 to 32.56 h, Altan et al. 2017). The t_1/2ʎz_ is a hybrid parameter related to the of Cl and V_d_ (Toutain and Bousquet‐mélou 2004). The prolongation of t_1/2ʎz_ in the LPS+CFQ+MLX group may be due to the changes in Cl_T_ and V_dss_.

The AUC was higher in the CFQ+MLX and LPS+CFQ+MLX groups than in the CFQ group. The MRT values showed significant differences between groups in the LPS+CFQ+MLX > CFQ+MLX > CFQ order. It has been reported that the AUC and MRT values of antibiotics increase in combination with meloxicam or in cases of endotoxemia (Elmas et al. 2006; Ural and Uney 2021; Waxman et al. 2003).

*E. coli, P. multocida, K. pneumoniae*, and *M. haemolytica* are bacterial pathogens responsible for causing septicaemia in livestock (Radostits et al. 2006). The MIC values for the *E. coli, P. multocida, K. pneumoniae*, and *M. haemolytica* strains isolated from sheep were below the susceptible breakpoint (≤ 2 µg/mL, Böttner et al. 1995; AVID 1999) of cefquinome (Table 3). The MIC values for *E. coli, M. haemolytica, and K. pneumonia* ranged from 0.031 to 0.063 µg/mL, whereas for *P. multocida* it measured between 0.016 and 1 µg/mL. The MIC value of cefquinome for bacteria isolated from sheep was not determined in any other study. MIC values of cefquinome for bacteria isolated from different animal species have been reported as 0.015‐2 µg/mL for *E. coli* (Chin et al. 1992; Thomas et al. 2006), ≤ 0.03‐0.5 µg/mL for *K. pneumonia* (Chin et al. 1992; Deshpande et al. 2000), ≤ 0.06‐4 µg/mL for *P. multocida* and *M. haemolytica* (Böttner et al. 1995). The MIC values determined in these studies are similar to the results in our study.

The effect of cefquinome is time‐dependent, and the T > MIC parameter is used to evaluate its antibacterial effect (Turnidge 1998). T > MIC values of 40% are desired for treatment success (Toutain et al. 2002). At a 12 h dosing interval, the CFQ, CFQ+MLX, and LPS+CFQ+MLX groups attained a T > MIC ratio of 40% for bacteria with MIC values of ≤ 0.50, ≤ 1, and ≤ 1 µg/mL, respectively. A T > MIC ratio of 40% was attained for bacteria with MIC values of ≤ 1, ≤ 2, and ≤ 2 µg/mL at the 8‐h dosing interval, respectively. However, T > MIC > 80% (Toutain et al. 2002) or T > 4 × MIC 100% (Mouton and Vinks 2007) is recommended in critically ill patients and immunocompromised patients. At a 12 h dosing interval, the CFQ+MLX and LPS+CFQ+MLX groups attained a T > MIC ratio of 80% for bacteria with MIC values of ≤ 0.12 and ≤ 0.5 µg/mL, respectively. The CFQ group did not reach this value in the 12 h dosing interval. At an 8 h dosing interval, the CFQ, CFQ+MLX, and LPS+CFQ+MLX groups attained a T > MIC ratio of 80% for bacteria with MIC values of ≤ 0.12, ≤ 0.50, and ≤ 1 µg/mL, respectively. These results indicate that the therapeutic effect of cefquinome may be increased when administered in combination with meloxicam in endotoxemic sheep.

## 5 Conclusion

Meloxicam and LPS+meloxicam administration decreased the elimination of cefquinome and increased its residence time in the body. In the 12 h dosing interval, the 80% T > MIC value was not achieved in the cefquinome alone group, while it was achieved in the CFQ+MLX and LPS+CFQ+MLX groups for bacteria with MIC values of ≤ 0.12 and ≤ 0.5 µg/mL, respectively. According to these results, the combined administration of meloxicam may increase the therapeutic efficacy of cefquinome. However, a detailed investigation into the therapeutic efficacy of cefquinome after combined application with meloxicam in naturally infected septicaemic sheep remains necessary.

## Author Contributions

**Muhittin Uslu**: conceptualisation, investigation, methodology, resources, supervision, project administration, writing ‐ original draft, writing ‐ review and editing. **Orhan Corum**: conceptualisation, investigation, methodology, resources, supervision, project administration, writing ‐ original draft, writing ‐ review and editing. **Enver Yazar**: conceptualisation, investigation, methodology, resources, supervision, project administration, writing ‐ original draft, writing ‐ review and editing.

## Ethics Statement

The experimental procedures used in the current experiment were approved by the Selcuk University Faculty of Veterinary Medicine Ethics Committee on 2023/12 (Konya/Türkiye).

## Conflicts of Interest

The authors declare no conflicts of interest.

## Peer Review

The peer review history for this article is available at https://www.webofscience.com/api/gateway/wos/peer‐review/10.1002/vms3.70462.

## Data Availability

All data are presented in manuscript itself. Further details may be obtained through mail muhittin.uslu@bozok.edu.tr
